# Predictive value of CCL2 in the prognosis and immunotherapy response of glioblastoma multiforme

**DOI:** 10.1186/s12864-023-09674-x

**Published:** 2023-12-06

**Authors:** Longfei Deng, Jie Ren, Benqin Li, Yinggang Wang, Nianfen Jiang, Yi Wang, Hongjuan Cui

**Affiliations:** 1 Cancer Center, Medical Research Institute, Southwest University, Chongqing, 400715 China; 2 Health Management Center, Southwest University Hospital, Chongqing, 400715 China; 3 Department of Endocrinology, The Ninth People’s Hospital of Chongqing, Chongqing, 400799 China; 4 State Key Laboratory of Silkworm Genome Biology, Southwest University, Chongqing, 400715 China

**Keywords:** C-C motif chemokine ligand 2, Glioblastoma multiforme, Prognosis, Immunotherapy, Prediction, Bioinformatic analysis

## Abstract

**Background:**

Glioblastoma multiforme (GBM) is the most common and lethal primary brain tumor with a poor prognosis. The C-C motif chemokine ligand 2 (CCL2) has shown abnormal expression associated with progression of multiple malignancies, however, its role in predicting the prognosis and immunotherapy response of GBM remains poorly understood.

**Results:**

CCL2 was highly expressed in GBM as analyzed by integrating CGGA, GEPIA and UALCAN online platforms, and further verified by histologic examinations, qRT-PCR analysis, and independent GEO datasets. CCL2 could serve as an independent prognostic factor for both the poor overall survival and progression-free survival of GBM patients based on TCGA data, univariate and multivariate cox analyses. Functional enrichment analysis revealed that CCL2 mainly participated in the regulation of chemokine signaling pathway and inflammatory response. Further, CCL2 expression was positively correlated with CD4 T cells, macrophages, neutrophils and myeloid dendritic cells infiltrating GBM as calculated by the TIMER2.0 algorithm. Importantly, the tumor immune dysfunction and exclusion (TIDE) algorithm showed that in CCL2-high GBM group, the expression of CD274, CTLA4, HAVCR2 and other immune checkpoints were significantly increased, and the immune checkpoint blockade (ICB) therapy was accordingly more responsive.

**Conclusions:**

CCL2 can be used as a predictor of prognosis as well as immunotherapy response in GBM, offering potential clinical implications.

**Supplementary Information:**

The online version contains supplementary material available at 10.1186/s12864-023-09674-x.

## Introduction

Glioblastoma multiforme (GBM) is the most common primary brain malignancy in adults, with an annual incidence rate of 3.21 cases per 100,000 people [1]. GBM is also the most aggressive brain tumor and the leading cause of cancer-related death worldwide, and the 5-year overall survival rate is only 5.6% after diagnosis [2]. With the rapid progress of high-throughput sequencing technology, some biomarkers of GBM have been uncovered, including IDH 1/2 mutation, TERT promoter mutation, 1p/19q co-deletion and others [3]. However, these biomarkers have limited value in predicting the survival of GBM patients in clinical practice. Therefore, identifying new biomarkers is crucial for GBM prognosis as well as therapeutic target discovery.

Currently, the standard option for treating GBM remains maximal surgical resection followed by adjuvant chemoradiotherapy [2]. Despite great progress achieved in developing novel treatment modalities for GBM, such as anti-tyrosine kinase receptor targeted therapies, most attempts have failed to yield significant clinical benefits [4]. On the other hand, the immunotherapy, represented by strategy of immune checkpoint inhibition (ICB), is emerging to be a revolutionized therapeutic for some solid malignancies [5]. Regardless of remarkable effects demonstrated in GBM cell lines in vitro, ICB therapies based on targeting PD-1, PD-L1, and CTLA4 have failed to improve patient survival in clinical trials, possibly in part due to the strong heterogeneity and immune evasion characteristic of GBM [6]. Although some candidate biomarkers, such as PD-L1 and IFN-γ, hold reference value in clinic decision of prescribing ICB therapies, these approaches are limited by degree of accuracy and scope of applications [7]. It is thus urgent to seek new potential biomarkers capable of gauging response to immunotherapy for GBM patients.

The C-C motif chemokine ligand 2 (CCL2) is a member of the CC chemokine family [8]. The abnormal expression of CCL2 has been reported in a variety of cancers, such as breast cancer, lung cancer and gastric cancer [9]. It is known that CCL2 secreted by tumor and stromal cells can recruit immune cells, including macrophages and peripheral leukocytes, to shape the tumor immune microenvironment (TIME) [10], a key factor determining the efficacy of ICB therapy [11]. However, the role of CCL2 in GBM prognosis and prediction of response to immunotherapy is not fully clarified. This study investigated the expression alteration and prognostic significance of CCL2 in GBM, and also assessed the predictive value of CCL2 in immunotherapy response, in an attempt to provide relevant theoretical basis for the prognosis and immunotherapy of this highly heterogeneous and fatal brain cancer.

## Materials and methods

### Cell line and culture

Human GBM cell lines (U87 MG, LN229, A172 and U251 MG) and human astroglial cell line SVGP12 were obtained from the American Type Culture Collection (ATCC) and authenticated by short-tandem repeat analysis [12]. All cell lines were cultured in a DMEM medium with 10% fetal bovine serum (FBS) in a humidified environment with 5% CO_2_ at 37 °C. These cell lines were confirmed mycoplasma-free by using a Mycoplasma Stain Assay Kit (C0296, Beyotime, China) according to manufacturer’s instructions.

### CCL2 expression analysis and immunohistochemistry

The CGGA (http://www.cgga.org.cn/), GEPIA (http://gepia.cancer-pku.cn/index.html) and UALCAN (http://ualcan.path.uab.edu/) online platforms were utilized to compare CCL2 expression between normal brain tissue and GBM. GBM tissue microarray was purchased from Xi’an bioaitech Co., Ltd (N095Ct01, Xi’an, China) and analyzed by immunohistochemistry using an anti-CCL2 antibody (AF7437, Beyotime, China) according to microarray instruction and a standard protocol [13]. Briefly, paraffin-embedded GBM tissues were dewaxed and hydrated. Paraffin slices were put into citrate buffer (pH 6.0) and heated (95 °C/20 min) to facilitate antigen retrieval. Endogenous peroxidase activity was then blocked, and goat serum was applied to tissue sections to block nonspecific binding before incubation with CCL2 primary antibodies overnight at 4 °C. A horseradish peroxidase-linked secondary antibody was added for further incubation and stained with DAB. Following haematoxylin re-staining, hydrochloric acid acidification, ammonia anti-blue, dehydration, and sealing, photos were obtained with a microscope.

### qRT-PCR analysis

The mRNA level of CCL2 in GBM cells was determined by qRT-PCR analysis as described in detail in a previous study [14]. In brief, total RNA was extracted via the TRIzol reagent (Invitrogen, USA), and 2 µg of total RNA were reversely transcribed into cDNA. The SYBR qPCR SuperMix Plus was used for qRT-PCR (Novoprotein, China), and the specific primer pairs are shown in Table 1. Results were calculated using the ΔΔCt method with the ACTB gene serving as the internal control.

**Table 1:** RT-qPCR primer sequence

Gene	Forward 5’-3’	Reverse 5’-3’
CCL2	ACCATTGTGGCCAAGGAGATCTGT	AGTTTGGGTTTGCTTGTCCAGGTG
ACTB	GACCTGACTGACTACCTCATGAAGAT	GTCACACTTCATGATGGAGTTGAAGG

### GEO dataset acquisition and analysis

According to the advanced retrieval of the GEO database (https://www.ncbi.nlm.nih.gov/gds/advanced), “glioblastoma multiforme” was input as keywords and the filter condition was set as “homo sapiens” in order to search for appropriate datasets of expression profiling by array. The inclusion criteria for GEO datasets was defined as follows: (1) samples were derived from clinical GBM tissue; (2) normal brain tissue should be contained as a control group; (3) expression profiling was mRNA expression array; (4) information of sequencing platform, gene ID and annotation should be completely included. The retrieved GEO datasets were analyzed via using the GEO2R (https://www.ncbi.nlm.nih.gov/geo/geo2r/) tool. The gene expression matrix file and corresponding gene annotation were downloaded. The differentially expressed genes (DEGs) were determined with a cut-off (adjusted *p* < 0. 05, |log_2_FC| > 1). Gene expression information of a matrix file was converted into a volcano plot and heatmap via OmicStudio (https://www.omicstudio.cn/tool) platform.

### Prognostic analysis

High-throughput RNA sequencing data of GBM samples and clinical information of corresponding patients were downloaded from TCGA database (https://portal.gdc.cancer.gov/). The survival differences between CCL2-low and CCL2-high expression groups were compared by the log-rank test, and the Kaplan-Meier survival curves were plotted. The risk score was calculated by the following formula:

$$\frac{{\sum\nolimits_{i = 1}^n {Ex{p_{i*}}} \frac{{ \pm 1}}{{{D_i}}}}}{{\sum\nolimits_{i = 1}^n {\frac{1}{{{D_i}}}} }}$$

In this formula, Exp represents CCL2 expression, ± is the positive or negative sign for the regression coefficient of CCL2 calculated by LASSO method, and D represents the variance in CCL2 expression in all the samples. Hazard ratios (HR) and their 95% confidence intervals (CI) were calculated using the estimated regression coefficients and their standard errors in Cox regression analysis.The variables with prognostic significance were calculated by the univariate and multivariate cox regression analyses, and the nomogram prognostic models were developed.

### Functional enrichment analysis

The gene ontology (gene ontology, GO) and kyoto encyclopedia of genes and genomes (KEGG) functional enrichment analyses of CCL2-related genes in GBM were performed using the David bioinformatics resources online platform (https://david.ncifcrf.gov/). Based on the expression profile of GEO matrix, the pathway enrichment of CCL2 was analyzed via the GSEA software (v4.3.2) (http://www.gsea-msigdb.org/gsea/index.jsp). A protein-protein interaction network (PPI) was constructed from the hub module through the Cytoscape software (v3.8.0) (https://cytoscape.org/), and validation of hub genes was performed using the MCODE and CytoHubba plugins.

### Immune score and correlation analysis

Based on TCGA database (https://portal.gdc.cancer.gov/), the immune score was calculated with an “immunedeconv” package of R software integrated with the TIMER2.0 algorithm [15, 16]. Correlations between gene expression and immune score were analyzed via a “ggstatsplot” package of R software [17].

### Immune checkpoint expression and ICB response analysis

Based on TCGA database (https://portal.gdc.cancer.gov/), the expression levels of 8 representative immune checkpoints in CCL2-low and CCL2-high expression GBM samples were compared, and the correlation between CCL2 and immune checkpoint expression was evaluated by the Spearman’s correlation analysis. The response to immune checkpoint blockade (ICB) therapy of CCL2-low and CCL2-high expression GBM groups was predicted with the tumor immune dysfunction and exclusion (TIDE) algorithm [18].

### Statistical analysis

Statistical analysis was performed with SPSS v.22.0 software (IBM Corp., Armonk, NY, USA) and GraphPad Prism v.7.0 software. R codes are accessible from the following references: R(v4.0.3) software package ggplot2(v3.3.3), pheatmap, ggpubr(0.4.0) (Core Team, 2020; Kassambara, 2020; Kolde and Kolde, 2018; Wickham, 2011). Survival curves were plotted using the Kaplan-Meier method. The significance of survival differences between CCL2-low and CCL2-high expression groups was compared by the log-rank test. The univariate and multivariate cox regression approaches were applied to analyze prognostic factors. Correlations were calculated with the Spearman’s correlation analysis. *p < 0. 05, ** *p* < 0.01, *** *p* < 0. 001 were considered statistically significant.

## Results

### CCL2 is highly expressed in GBM

**Fig. 1:**
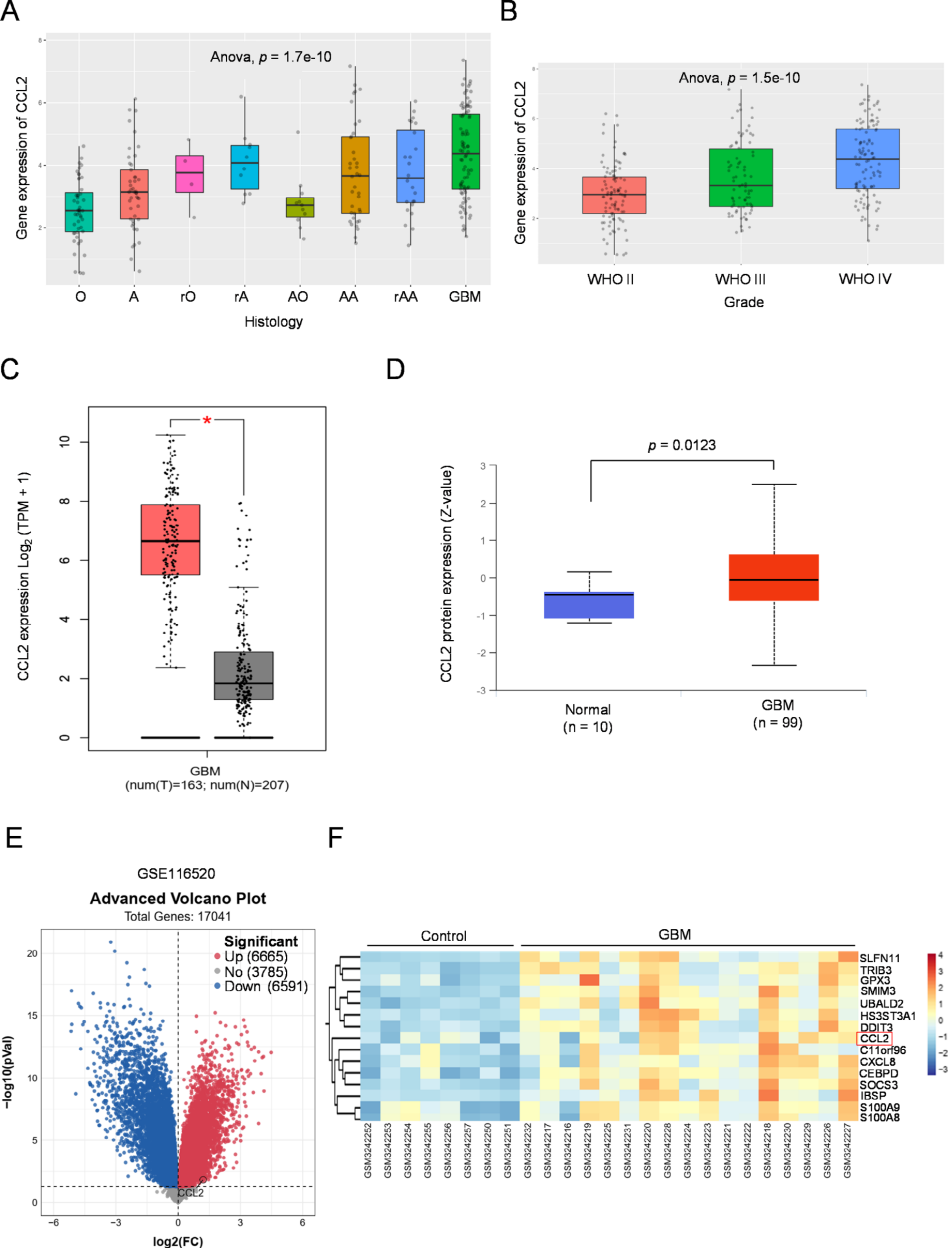
CCL2 is highly expressed in GBM. **A**, **B** The expression level of CCL2 in GBM (**A**) and gliomas stratified with different WHO grade (**B**) was analyzed with the mRNAseq_325 dataset in the CGGA atlas. O, oligoastrocytoma; A, astrocytoma, rO, recurrent oligoastrocytoma; rA, recurrent astrocytoma; AO, anaplastic oligoastrocytoma; AA, anaplastic astrocytoma; rAA, recurrent anaplastic astrocytoma; GBM, glioblastoma multiforme. *p* value, GBM vs. O (**A**) and WHO IV vs. III (**B**). **C** CCL2 expression in GBM and normal counterparts was compared using GEPIA datasets. TPM, transcripts per million. **D** Comparison of CCL2 protein level in GBM and normal counterparts was implemented using the CPTAC proteomic data. * *p* < 0.05, Wilcox test. **E**, **F** The volcano plot (**E**) and heatmap plot (**F**) of differentially expressed genes (DEGs) were derived from GSE116520 dataset

Analysis with the CGGA database (http://www.cgga.org.cn/) showed that CCL2 was significantly overexpressed in GBM among other histological types of brain tumors (Fig. 1A), and that its expression level was positively correlated with WHO grade of glioma (Fig. 1B), in which grade IV (GBM) displayed the highest level of CCL2, suggesting that CCL2 may be associated with malignant progression of glioma. Additionally, compared with normal group, higher expression of CCL2 in GBM was also obtained with the GEPIA platform (http://gepia.cancer-pku.cn/index.html) (Fig. 1C, Fig. S1). In addition to mRNA upregulation, the UALCAN (http://ualcan.path.uab.edu/) online tool revealed that higher CCL2 protein level was expressed in GBM (Fig. 1D). To verify these results, we acquired and analyzed three independent GEO datasets (GSE116520, GSE104267, and GSE12657) which contained mRNA profiling of human GBM tissue. The results showed that CCL2 was indeed highly expressed in GBM compared with normal brain tissue, as shown in datasets of GSE116520 (Fig. 1E-F), GSE104267 (Fig. S2A-B), and GSE12657 (Fig. S2C-D). Hence, these data suggest that CCL2 is highly expressed in GBM.

### Experimental validation of high CCL2 expression in GBM

**Fig. 2:**
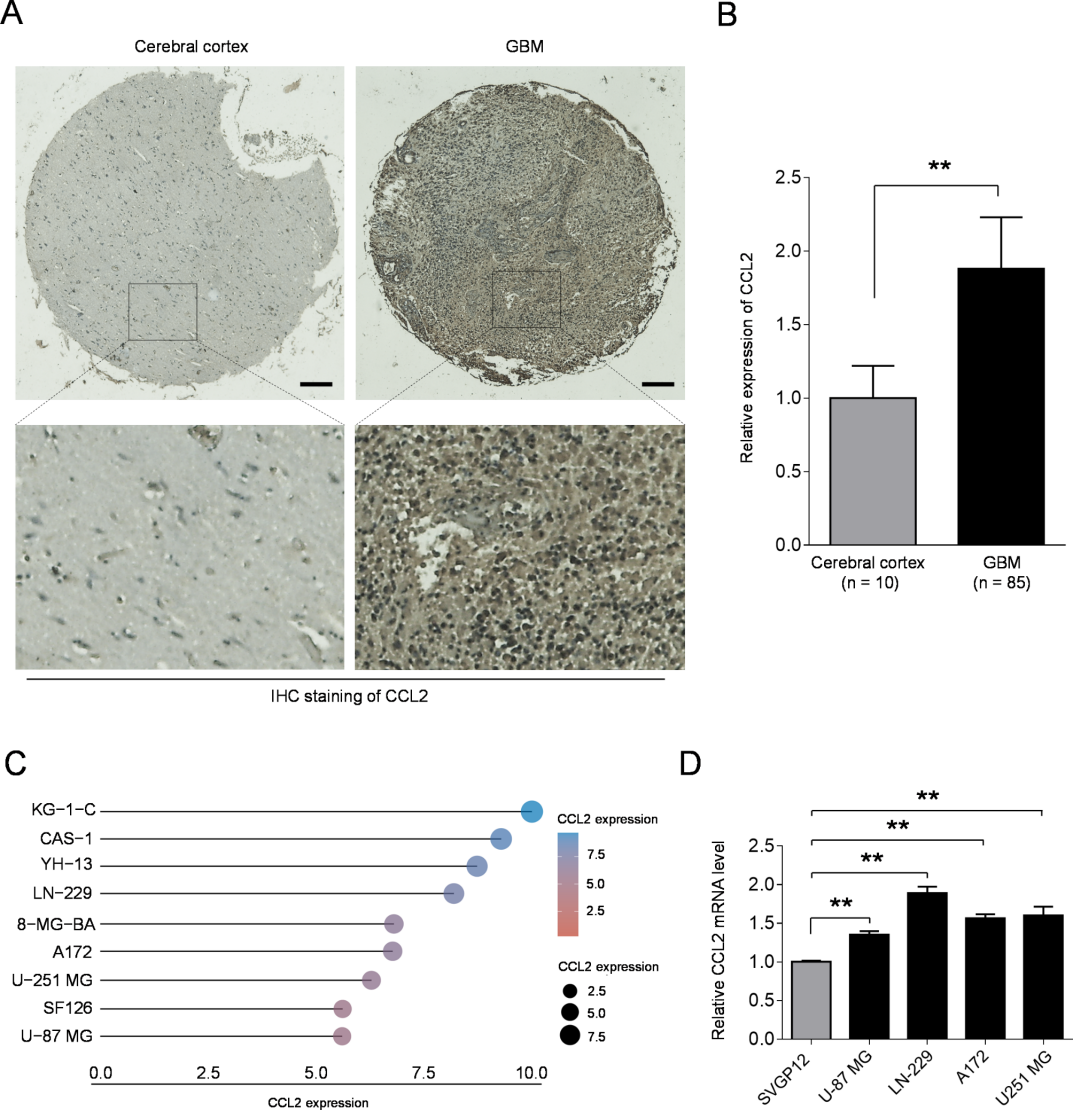
Experimental validation of high CCL2 expression in GBM. **A**, **B** Immunohistochemistry (IHC) staining of CCL2 with a GBM tissue microarray. The normal brain tissue was used as a control. Scale bar, 200 µM. **C** CCL2 expression in representative GBM cell lines obtained from the CCLE dataset. **D** The transcript level of CCL2 in 4 available GBM cell lines and a normal brain glial SVGP12 cells was detected by qRT-PCR analysis. Data are the mean ± s.d (n = 3). Student’s t-test. ** *p* < 0.01

To consolidate the above analyses, we checked the expression of CCL2 with a GBM tissue microarray through immunohistochemistry (IHC) experiment. Consistently, the results exhibited that CCL2 was overexpressed in GBM compared to normal brain tissue (Fig. 2A-B; Table 2). Interestingly, a large-scale profiling of cell line using the Cancer Cell Line Encyclopedia (CCLE) (https://portals.broadinstitute.org/ccle) also showed that CCL2 was expressed with a high level in GBM cell lines across many cancer types (Fig. 2C, Fig. S3) [19]. To check whether CCL2 is overexpressed in GBM cells in vitro, we measured CCL2 expression with 4 available GBM cell lines by qRT-PCR analysis. As a result, we detected higher transcript level of CCL2 in theses GBM cells lines, including U-87 MG, LN-229, A172 and U251 MG, as compared with the normal brain glial SVGP12 cells (Fig. 2D; Table 1). Altogether, these experimental validations demonstrate that CCL2 is highly expressed in GBM.

**Table 2:** CCL2 expression in normal cerebral cortex and GBM tissues

Tissue	High expression cases	High expression rate (%)	*p* value
Cerebral cortex	1	10	< 0.01
GBM	52	61	

### CCL2 is an independent prognostic factor for GBM

**Fig. 3:**
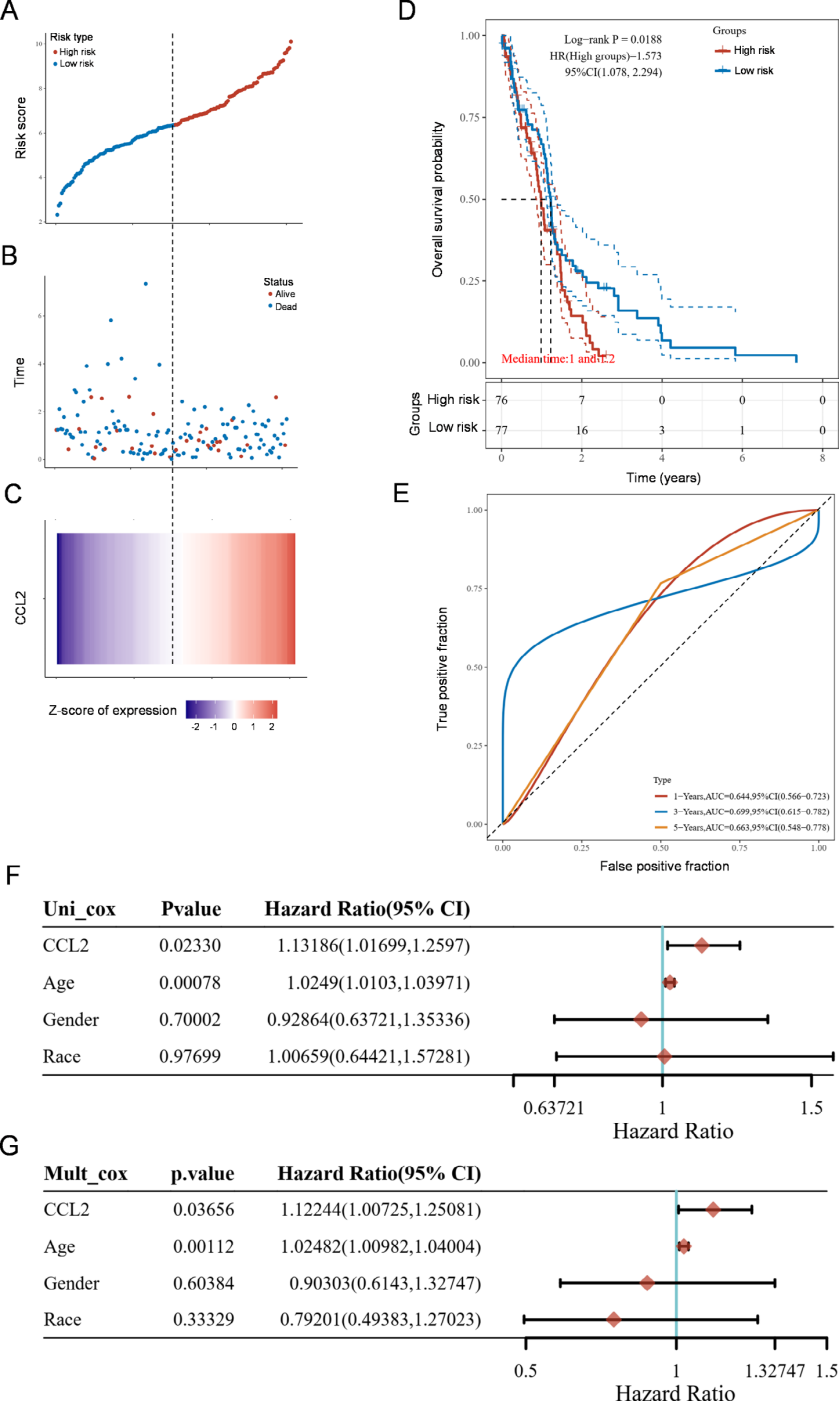
CCL2 is an independent prognostic factor for the overall survival of GBM. **A** Distribution of the risk score in the TCGA training set. **B** The survival time and survival status between high- and low-risk groups. **C** Heatmap of the expression profiles of CCL2 in low- and high-risk groups. The dotted line indicated the median risk score and divided the cohort into low- and high-risk group. **D** Kaplan-Meier survival analysis of the overall survival (OS) of GBM patients in the high- and low-risk groups. **E** Time-dependent ROC analysis of the predictive efficiency of CCL2 on the 1-, 3-, and 5-years OS rate. **F**, **G** Univariate (**F**) and multivariate (**G**) cox analyses evaluating the independent prognostic value of CCL2 in OS of GBM patients

To evaluate whether CCL2 has prognostic value in GBM patients, high-throughput RNA sequencing data and corresponding survival status of GBM patients were downloaded from the TCGA database (https://portal.gdc.cancer.gov/), which manifested that high CCL2 expression was a risk factor (Fig. 3A-C). The Kaplan-Meier curves, in which *p* values and hazard ratio (HR) with 95% confidence interval (CI) were generated by the log-rank test and univariate cox proportional hazards regression, displayed that the high-risk group had worse overall survival (OS) compared with the low-risk group (Fig. 3D). The area under an ROC curve (AUC) of the time-dependent ROC analysis was 0.644, 0.699 and 0.663 for 1-year, 3-year, and 5-year OS, respectively (Fig. 3E). The nomogram models developed with univariate analysis (Fig. 3F) and multivariate cox regression analysis (Fig. 3G) of CCL2 expression and other clinical characteristics including age, gender and race depicted that CCL2 was a significant independent factor capable of predicting OS. Moreover, we also assessed the predictive value of CCL2 in the progression free survival (PFS) of GBM patients, and similar results were obtained (Fig. S4). Collectively, these lines of evidence indicate that CCL2 could serve as an independent factor for GBM prognosis.

### Functional analysis of CCL2 in GBM

**Fig. 4:**
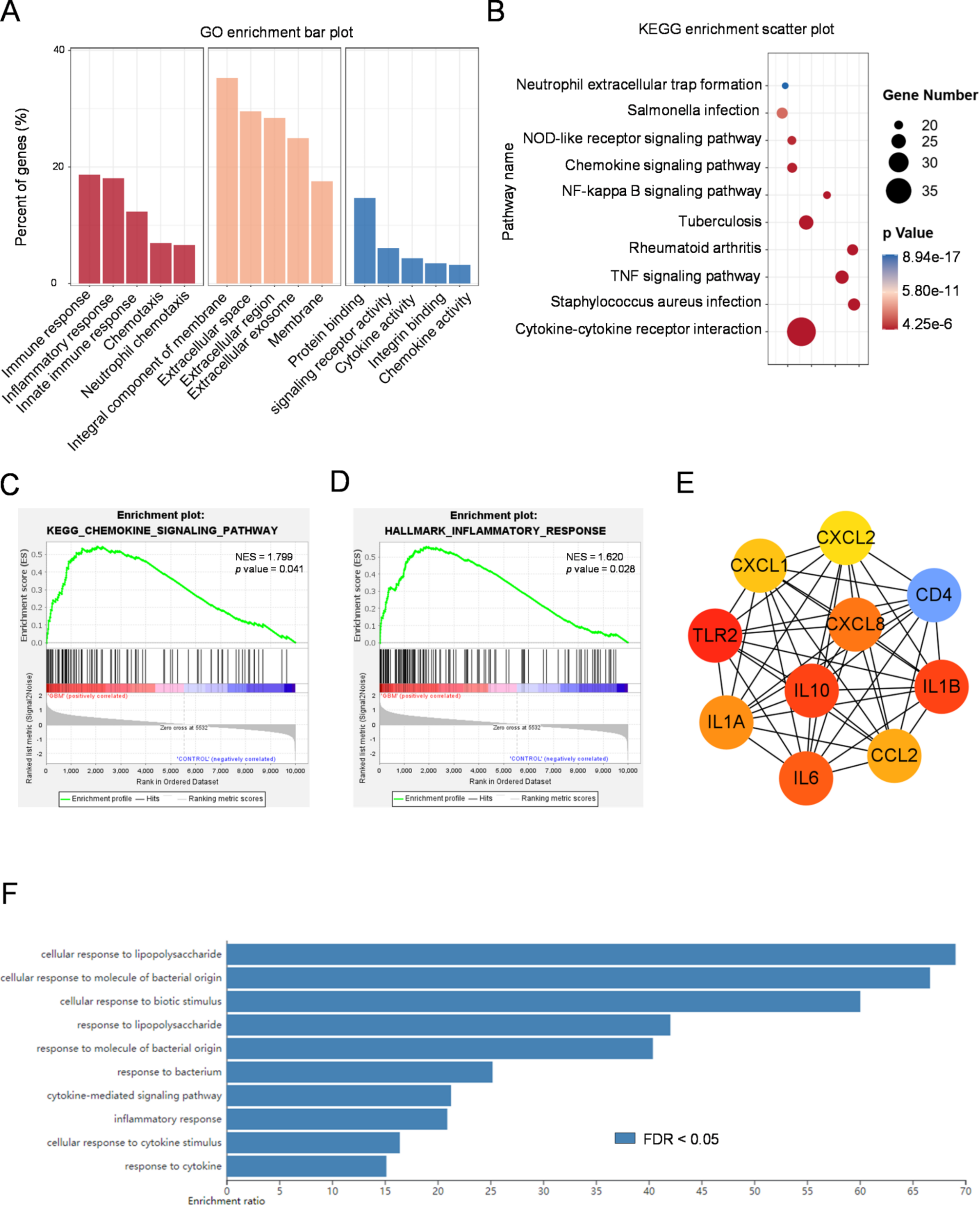
Functional analysis of CCL2 in GBM. **A**, **B** The gene ontology (GO) (**A**) and kyoto encyclopedia of genes and genomes (KEGG) (**B**) analyses of 370 genes which are most positively associated with CCL2 in GBM in the TCGA dataset. **C**, **D** The gene set enrichment analysis (GSEA) of GSE104267 dataset showing that CCL2 was enriched in chemokine signaling pathway (**C**) and inflammatory response (**D**) in GBM. **E** The 10 hub genes associated with CCL2 were constructed by Cytoscape software. **F** Functional analysis of biological process of 10 hub genes was performed using the online toolkit WebGestalt.

To understand the potential biological roles and related pathways of CCL2 involved in GBM pathogenesis, we took advantage of the TCGA data and analyzed the correlation between CCL2 and other genes in GBM, and then performed functional enrichment analysis of the top 370 genes which were most positively associated with CCL2 (*p* < 0.05, Spearman’s correlation coefficient > 0.5, Fig. S5A-C, Table S1). The gene ontology (GO) analysis showed that the major roles of these CCL2-associated genes were linked to immune and inflammatory responses, signal transduction and chemotaxis (Fig. 4A). Besides, the kyoto encyclopedia of genes and genomes (KEGG) pathway analysis indicated that these 370 genes were functionally enriched in some inflammatory and chemokine signaling pathways and inflammation associated diseases (Fig. 4B). To strengthen the above results, we conducted the gene set enrichment analysis (GSEA) of a GEO dataset. In concert, the results also pointed out that compared with normal brain tissue, genes upregulated in GBM including CCL2 were significantly enriched in chemokine signaling pathway (Fig. 4C) and inflammatory response (Fig. 4D). To further identify hub genes among these CCL2-related genes, we constructed a protein-protein interaction network (PPI) through the Cytoscape software (Fig. S7A) and 10 hub genes including CCL2 were obtained (Fig. 4E). Analysis of these 10 hub genes using the online toolkit WebGestalt (http://webgestalt.org/) also showed a primary functional participation in immune response mediated by cytokines and chemokines (Fig. 4F and Fig. S7B). It is worth to mention that all these 10 hub genes were highly expressed in GBM (Fig. S5D), and that similar to CCL2, a high expression of this 10-gene signature was significantly associated with poor OS prognosis in patients with GBM (Fig. S5E-I). These clues hint that the upregulated expression of CCL2 and related gene network may play a role in GBM progression.

### Correlation between CCL2 and immune cells infiltrating GBM

**Fig. 5:**
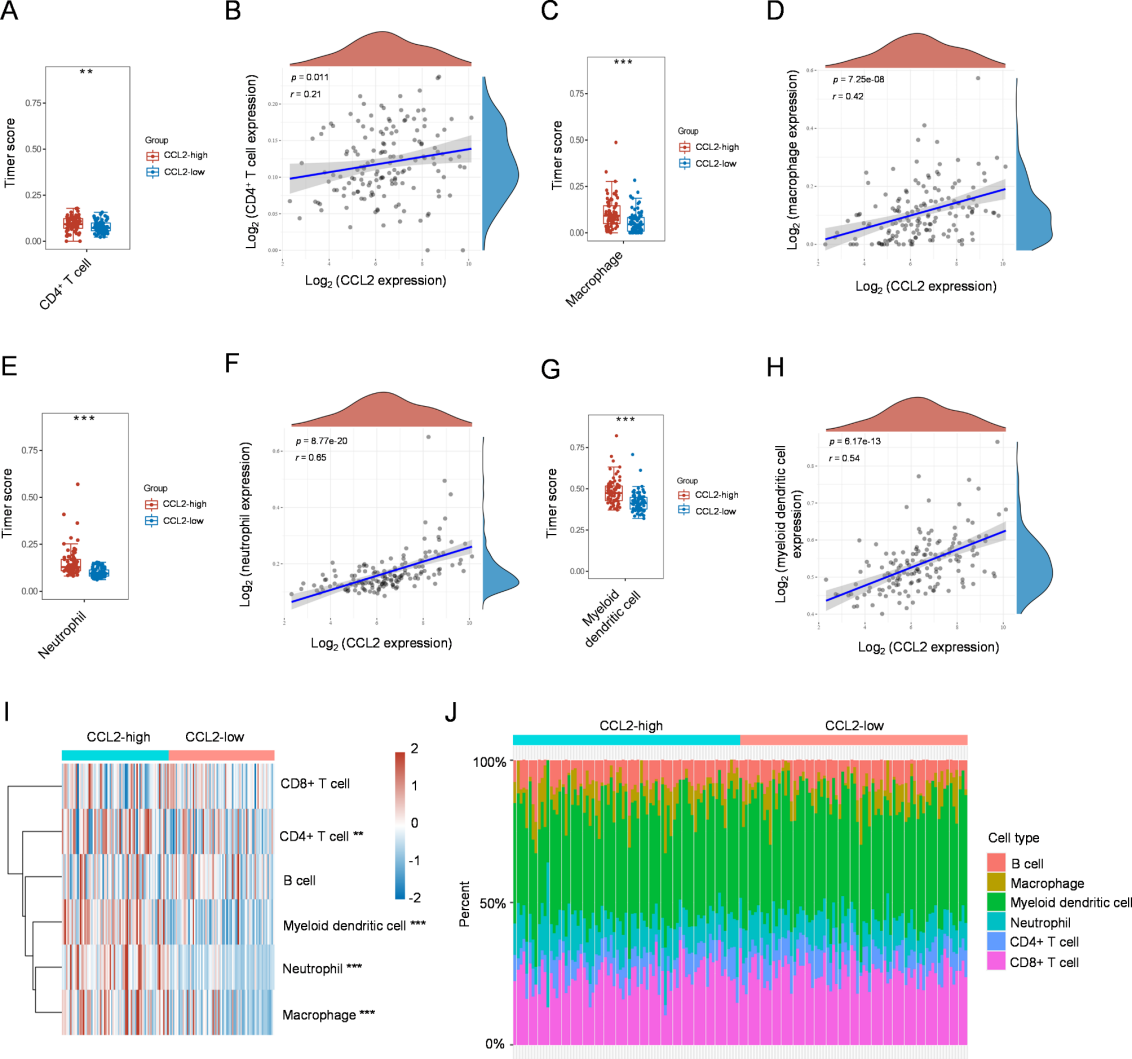
Correlation between immune cell infiltration and CCL2 in GBM. **A** Immune cell score of CD4^+^ T cells in CCL2-high and CCL2-low GBM tissue was calculated with the TIMER2.0 algorithm. **B** The correlation between CCL2 expression and immune score of CD4^+^ T cells was analyzed with Spearman’s correlation analysis. **C**-**H** Similar analyses for macrophages (**C**, **D**), neutrophils (**E**, **F**), and myeloid dendritic cells (**G**, **H**) were performed and results were displayed as in A and B. **I**, **J** The heatmap of immune cell score in CCL2-high and CCL2-low GBM tissue (**I**) and the percentage abundance of tumor infiltrating immune cells in each sample (**J**) were depicted. The statistical difference was calculated through the Wilcox test. ** *p* < 0.01, *** *p* < 0.001

In light of the above results, we postulated that CCL2 may affect the infiltration of immune cells in GBM. To test this possibility, we compared the differences of immune cells in CCL2-low and CCL2-high GBM groups, and also analyzed the correlation of CCL2 with a variety of immune cells through TIMER2.0 algorithm [16]. As expected, in CCL2-high GBM, the immune score and correlation of CD4^+^ T cells (Fig. 5A-B), macrophages (Fig. 5C-D), neutrophils (Fig. 5E-F), myeloid dendritic cells (Fig. 5G-H), and NK cells (Fig. S6E-F) were all significantly increased compared with CCL2-low GBM. Interestingly, however, we did not notice significant changes in other types of immune cells between these two groups, including B cells and CD8^+^ T cells (Fig. 5I-J, Fig. S6A-D). Nonetheless, these data suggest that CCL2 promotes the infiltration of certain types of immune cells into GBM tissue.

### CCL2 predicts response to ICB therapy in GBM patients

**Fig. 6:**
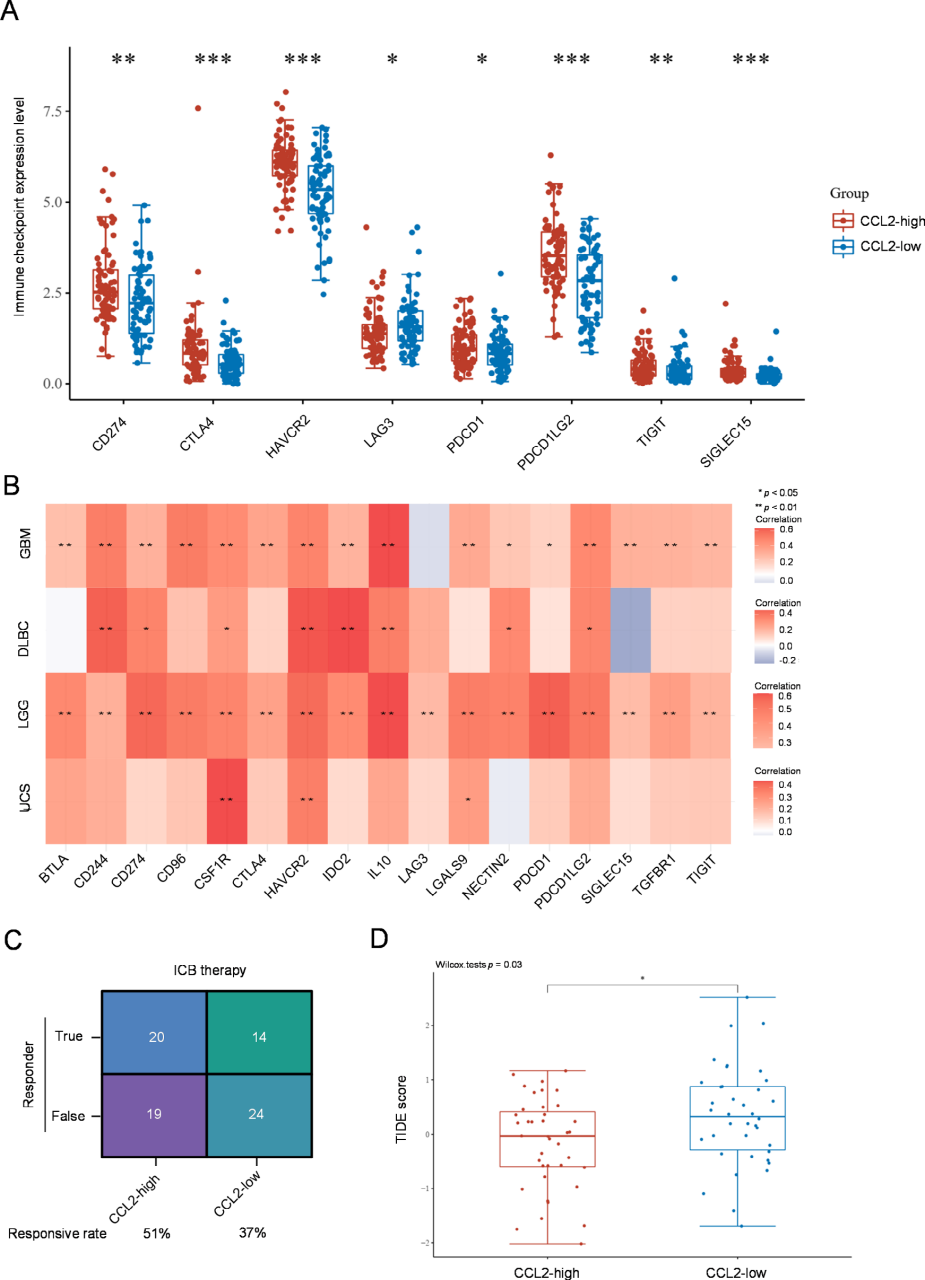
CCL2 predicts response to immune checkpoint blockade therapy. **A** The expression pattern of 8 immune checkpoint genes in CCL2-high and CCL2-low GBM tissue. The statistical difference was calculated through the Wilcox test. * *p* < 0.05, ** *p* < 0.01, *** *p* < 0.001. **B** A heatmap of the correlation between CCL2 and multiple immune checkpoint genes in GBM, DLBC, LGG and UCS, as analyzed by the Spearman’s correlation analysis. * *p* < 0.05, ** *p* < 0.01. DLBC, lymphoid neoplasm diffuse large B-cell lymphoma; LGG, brain lower grade glioma; UCS, uterine carcinosarcoma. **C** The number of patients responsive (true) or irresponsive (false) to the immune checkpoint blockade (ICB) therapy. **D** Boxplot representation of TIDE score in CCL2-high group versus CCL2-low in TCGA cohort. The statistical difference was calculated through the Wilcox test. * *p* < 0.05

The finding that CCL2 may influence the TIME of GBM prompts us to examine whether CCL2 is associated with the levels of immune checkpoints. To this end, we utilized TCGA dataset and compared the levels of 8 representative immune checkpoints in CCL2-low and CCL2-high GBM groups. We found that except LAG3, the levels of CD274, CTLA4, HAVCR2, PDCD1, PDCD1LG2, TIGIT and SIGLEC15 were all significantly increased in CCL2-high GBM group (Fig. 6A). Additionally, except these immune checkpoints, we also noticed that most of immune checkpoints were associated positively with CCL2 level in GBM (Fig. 6B), suggesting that high CCL2 expression may be tightly related to immune suppressive status in GBM. Based on these results reflecting that CCL2 affects the immune cell infiltration and levels of immune checkpoints, we further hypothesized that CCL2 might be a predictive factor for the immune checkpoint blockade (ICB) therapy. To demonstrate this idea, we applied the tumor immune dysfunction and exclusion (TIDE) algorithm to evaluate whether CCL2 can predict the response of GBM patients to ICB therapy [18]. The results indicated that compared to patients with CCL2-low GBM, ICB therapy produced a better therapeutic response in those with high CCL2 expression (Fig. 6C-D). Taken together, these findings propose that CCL2 could serve as a valuable predictive factor for ICB therapy response in GBM patients.

## Discussion

This study integrated multiple bioinformatic analyses and experimental validations and found that CCL2 was significantly overexpressed in GBM, and can be used as an independent prognostic factor to predict the OS and PFS of GBM patients. Functionally, CCL2 was mainly involved in the regulation of immune and inflammatory responses, and cell chemotaxis in GBM. In consistence, we also provided some lines of evidence suggesting that CCL2 was positively associated with CD4^+^ T cells, macrophages, neutrophils, myeloid dendritic cells, and NK cells infiltrating GBM, and that the expression levels of a majority of immune checkpoints were also positively associated with CCL2. Lastly, high CCL2 expression predicted a better response to ICB therapy in GBM patients. In conclusion, our results support CCL2 to be a potential prognostic factor for GBM and also a predictive biomarker for immunotherapy, providing a rationale for assisting individualized survival prediction and more precise selection of GBM patients who could benefit from immunotherapy.

In addition to GBM, we also found that the level of CCL2 was also elevated in lymphoid neoplasm diffuse large B-cell lymphoma (DLBC), brain lower grade glioma (LGG), and uterine carcinosarcoma (UCS) (Fig. S1), which is consistent with previous reports [20–22], suggesting that CCL2 may play a role in the pathogenesis of multiple cancers including GBM. In recent years, some studies have demonstrated that CCL2 can be considered as an independent prognostic factor for bladder cancer [23], clear-cell renal cell carcinoma [24], and gastric cancer [9], together with our findings, further highlighting a significant value of CCL2 in human cancer prognosis. CCL2 is an important CC chemokine family member participating in the regulation of directed migration and infiltration of reticuloendothelial cells, especially monocytes/macrophages, whereby affecting related immune responses [25]. It is also documented that CCL2 plays an important role in immune surveillance and immune response regulation in GBM [26]. Of note, in this study, we did not discover significant difference of other immune cells such as B cells and CD8^+^ T cells in CCL2-low and CCL2-high GBM (Fig. S6), implying that CCL2 imposes certain selective regulation on immune cell infiltration into GBM, however, the specific mechanisms require further investigations. It has been demonstrated that the expression of CCL2 is associated with polarization of CD4^+^ T cells and CCL2 secreted from the TME could promote their aggregation [27, 28]. Previous studies also have shown that CCL2 is able to promote tumor progression by recruiting and reprogramming tumor-associated macrophages, which accelerate tumor proliferation and metastasis by inducing immune escape [29, 30]. CCL2 was reported to mediate the recruitment of neutrophils, which are thought to facilitate tumor progression through CCL2 expression [31, 32]. In an autoimmune disease model, CCL2 expression results in accumulation of myeloid dendritic cells in the central nervous system [33]. Moreover, one study has illustrated that CCL2 is essential for NK cell recruitment, providing an insight into the design of effective NK cell-based therapies for cancer [34]. These lines of evidence support our observation of a positive correlation between CCL2 and CD4^+^ T cells, macrophages, neutrophils, myeloid dendritic cells and NK cells infiltrating GBM, suggesting that CCL2 may remodel the TIME of GBM.

The immune checkpoints are regulatory molecules that play an inhibitory role in the immune system and are essential for maintaining autoimmune tolerance. On the other hand, the immune checkpoints expressed on immune cells inhibit their function and disable the individual to produce effective anti-tumoral immune response, ultimately leading to tumor immune escape [35]. ICB therapy is a revolutionized approach used to treat cancer by harnessing the power of immune system [36]. Whereas, an apparent limitation for ICB therapy is that in most of the tested subjects, only a third of patients have responded to this therapy, in which the degree of infiltration of immune cells and the levels of immune checkpoints are among the crucial factors that are believed to influence ICB efficacy [37]. We found that CCL2 was associated positively with the infiltration of some immune cells and the level of immune checkpoints in GBM tissue, therefore providing at least one mechanistic explanation for the predictive role of CCL2 in ICB therapy response in GBM patients. Intriguingly, aside from GBM, we found that the levels of most immune checkpoints were positively associated with CCL2 in DLBC, LGG, and UCS as well (Fig. 6B). This seemingly suggests that CCL2 may also have potential predictive value in immunotherapy response in these cancers. More studies are needed in the future to test this possibility.

### Electronic supplementary material

Below is the link to the electronic supplementary material.

Supplementary Material 1

Supplementary Material 2

Supplementary Material 3

## Data Availability

The TCGA cohort data are publicly available and can be obtained from the accessible link of NCI’s Genomic Data Commons portal (https://portal.gdc.cancer.gov/). Three independent GEO datasets (GSE116520, GSE104267, GSE12657) are freely available for download via the GEO database (http://www.ncbi.nlm.nih.gov/geo/). All data and source data are included in the paper and/or the supplementary materials. Additional data are available upon request from corresponding authors.
