# Supplementary material for: Predictive value of CCL2 in the prognosis and immunotherapy response of glioblastoma multiforme
Source: BMC Genomics. 2023 Dec 6;24:746. doi: 10.1186/s12864-023-09674-x (PMC10698956; doi:10.1186/s12864-023-09674-x)
Supplement: Supplementary file 1 — Supplementary Material 1 [file 12864_2023_9674_MOESM1_ESM.pdf]

Fig S1

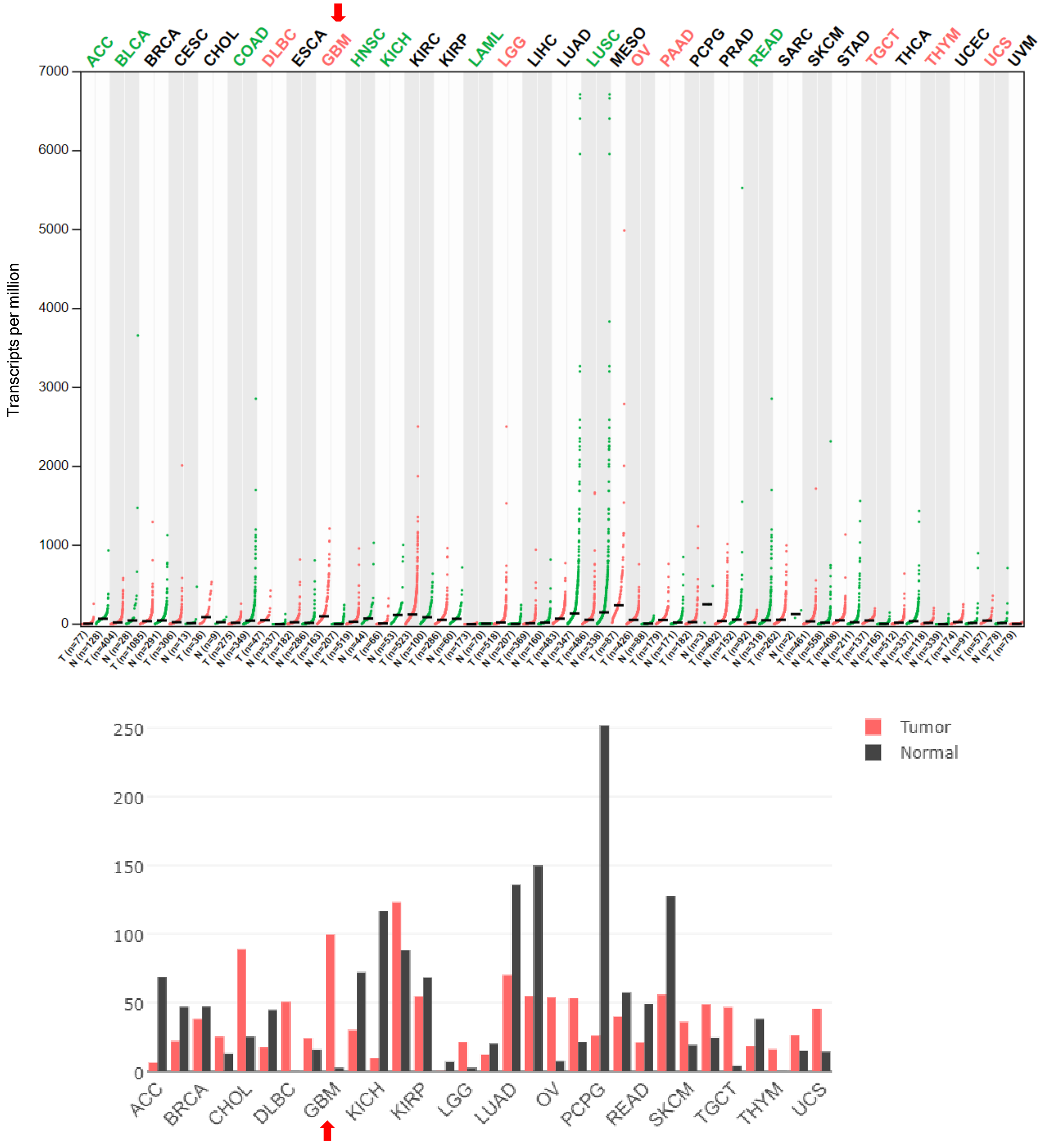

Fig. S1 CCL2 is highly expressed in GBM. The gene expression profile across all tumor samples and paired normal tissues was analyzed using GEPIA datasets. The cancer names marked in red color represent a significant upregulation of CCL2, and those marked in green color indicate a significant CCL2 downregulation in the corresponding tumors. Each dot represents the expression of a sample (dot plot). The height of bar represents the median expression of certain tumor type or normal tissue (bar plot).

Fig S2

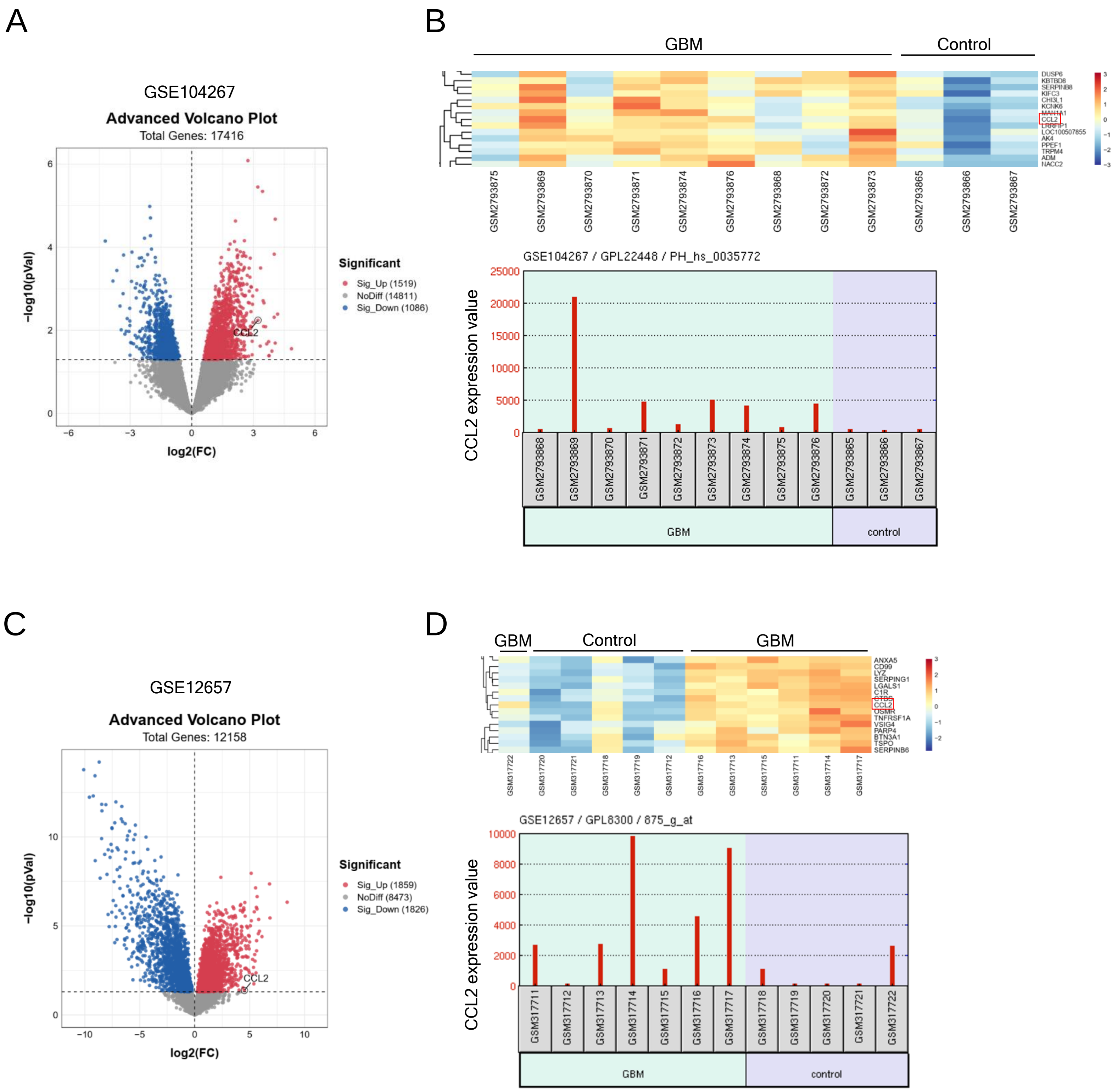

Fig. S2 Independent GEO datasets confirm high expression of CCL2 in GBM. **A**, **B** The volcano plot (**A**) and heatmap plot (**B**) of differentially expressed genes (DEGs) were derived from GSE104267 dataset, and CCL2 expression level in each sample was also shown below. Similar analyses for GSE12657 dataset (**C**, **D**) were performed and results were displayed as in A and B.

Fig S3

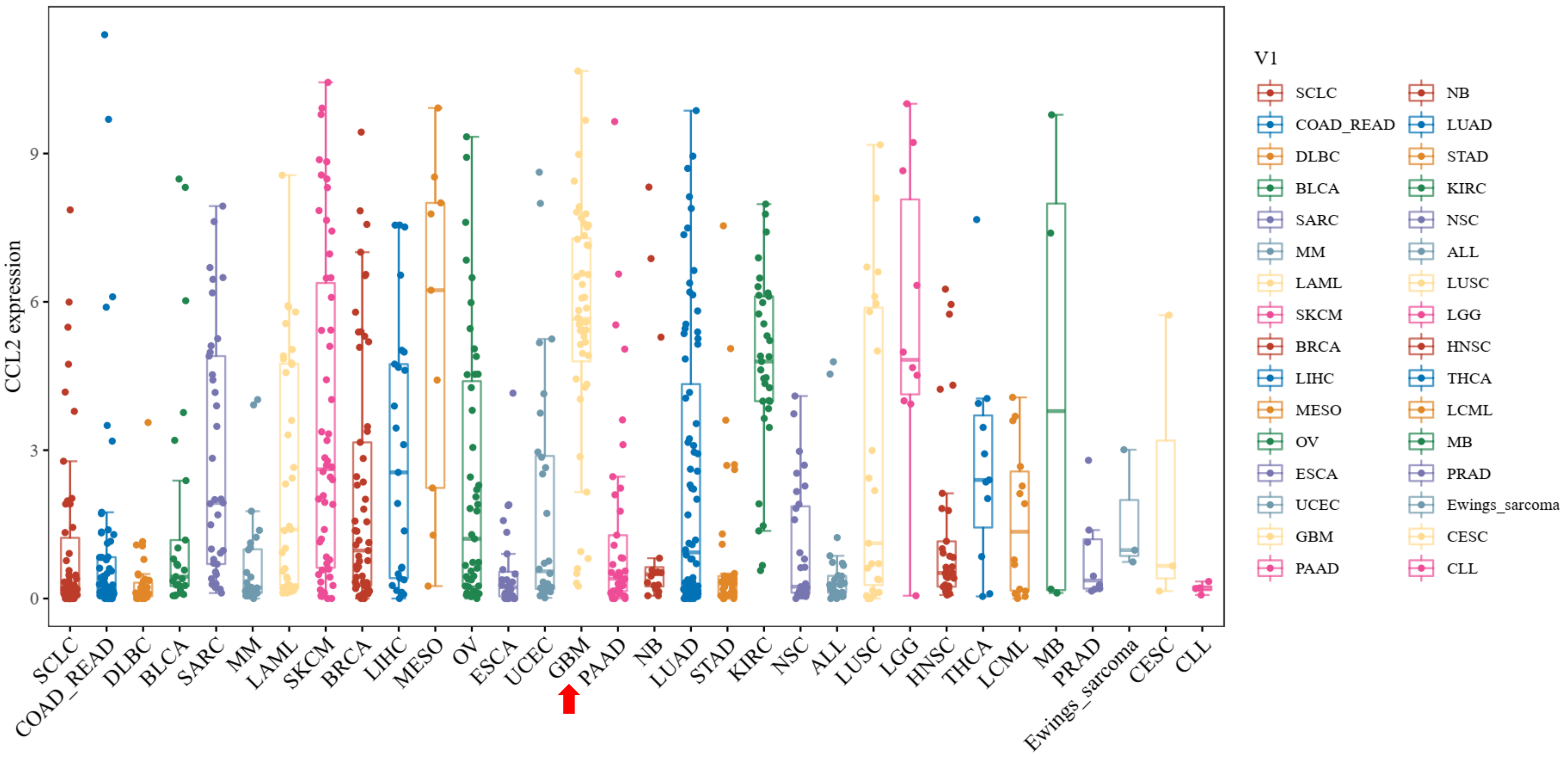

Fig. S3 High expression of CCL2 in GBM cell lines. The cell line mRNA expression matrix of tumors was obtained from the CCLE dataset (https://portals.broadinstitute.org/ccle). The analysis was constructed by the R v4.0.3 software package ggplot2 (v3.3.3).

Fig S4

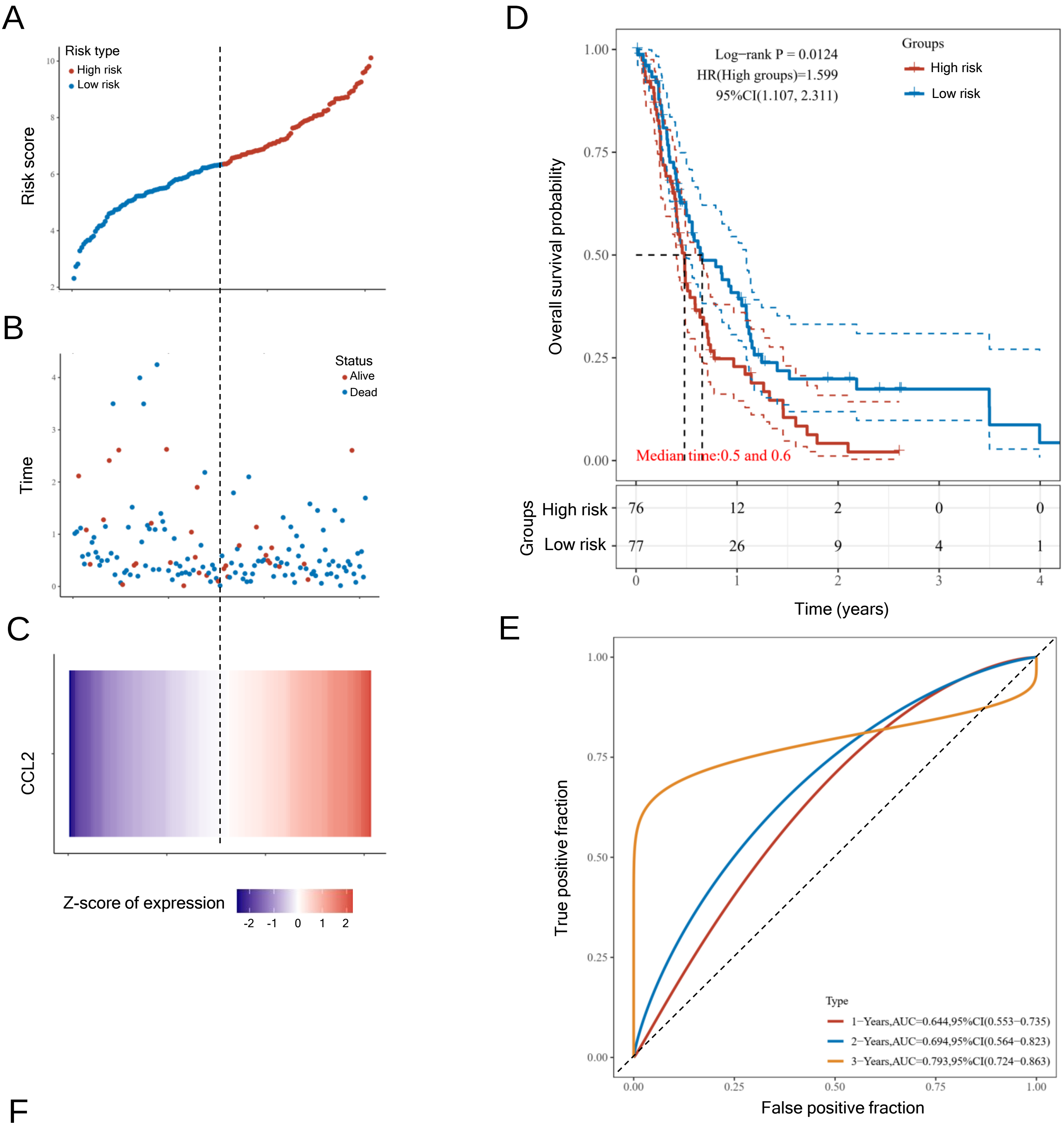

F

Uni_cox	Pvalue	Hazard Ratio(95% CI)
CCL2	0.00550	1.15739(1.04392,1.28319)
Age	0.05580	1.01306(0.99968,1.02662)
Gender	0.42315	1.16744(0.7993,1.70515)
Race	0.59152	0.88393(0.56326,1.38714)

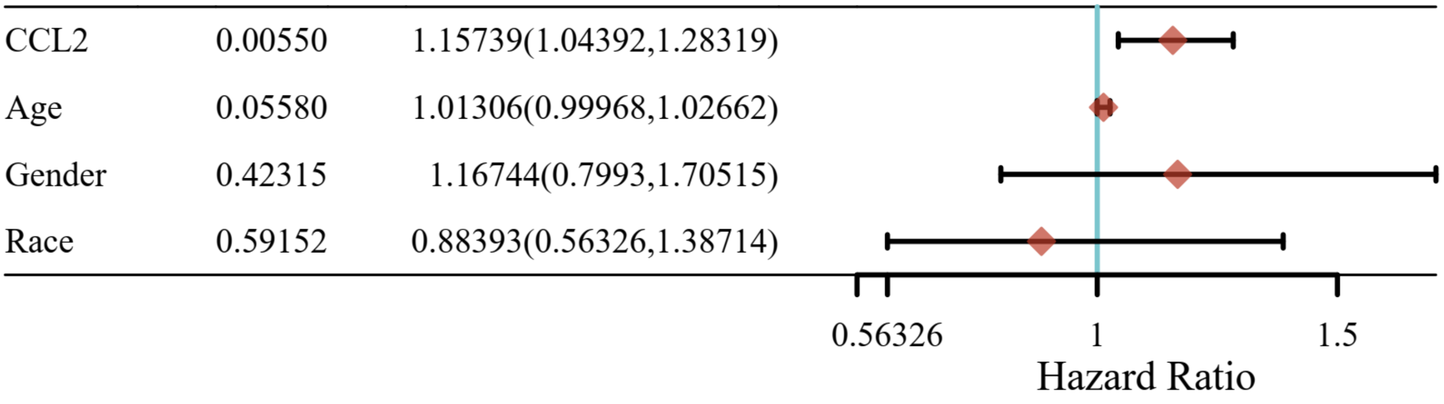

See next slide for figure legends

G

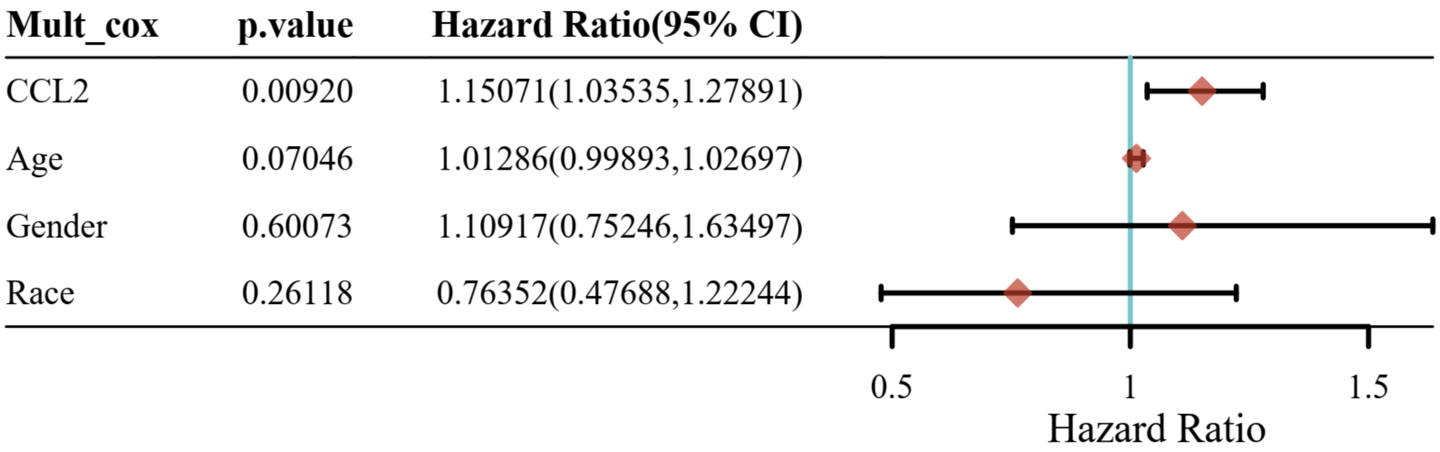

Fig. S4 CCL2 is an independent prognostic factor for the progression free survival of GBM. **A** Distribution of the risk score in the TCGA training set. **B** The survival time and survival status between high- and low-risk groups. **C** Heatmap of the expression profiles of CCL2 in low- and high-risk groups. The dotted line indicated the median risk score and divided the cohort into low- and high-risk group. **D** Kaplan-Meier survival analysis of the progression free survival (PFS) of GBM patients in the high- and low-risk groups. **E** Time-dependent ROC analysis of the predictive efficiency of CCL2 on the 1-, 3-, and 5-years PFS rate. **F**, **G** Univariate (**F**) and multivariate (**G**) cox analyses evaluating the independent prognostic value of CCL2 in PFS of GBM patients.

Fig S5

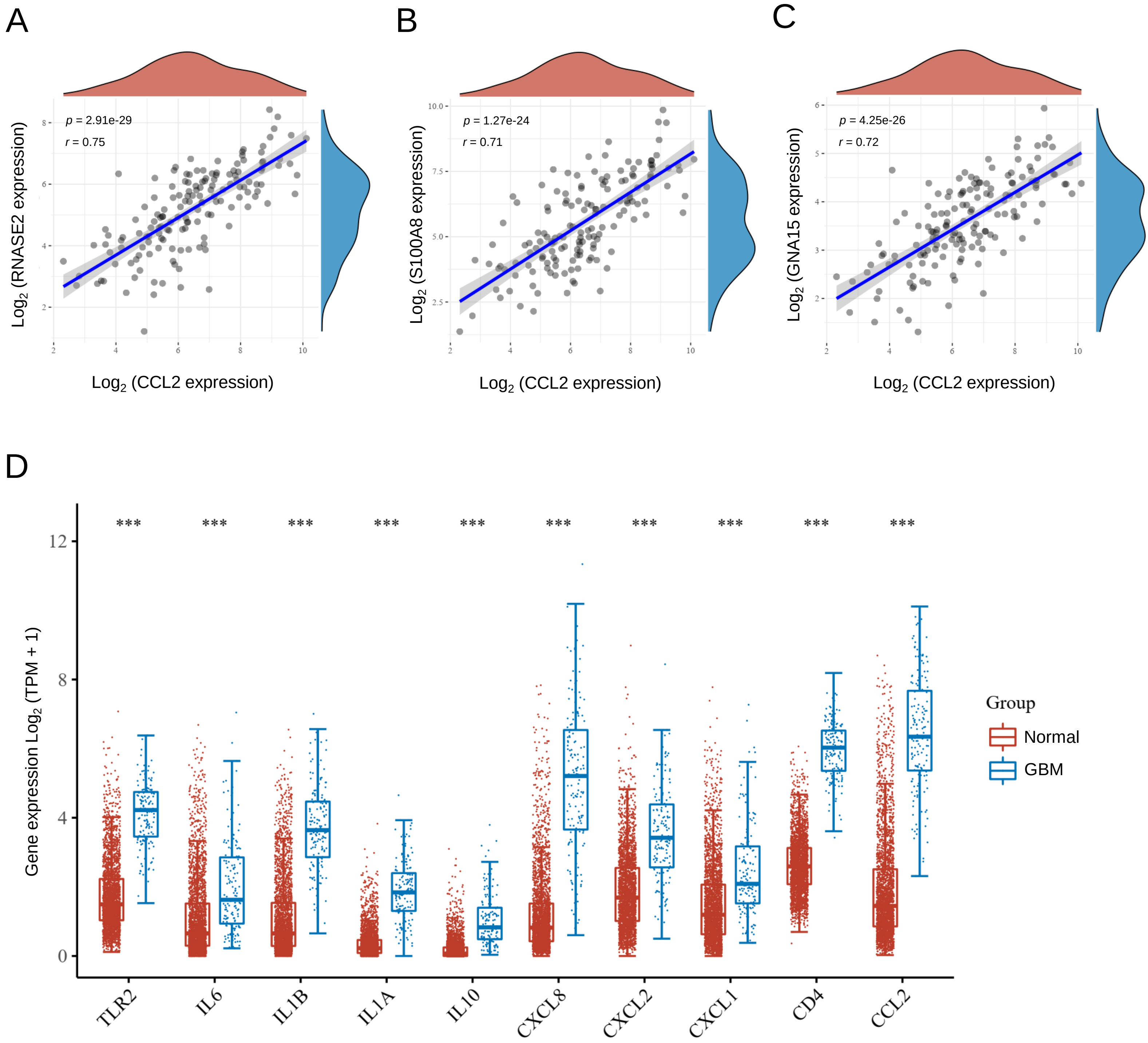

See next slide for figure E-I and figure legends

Fig S5

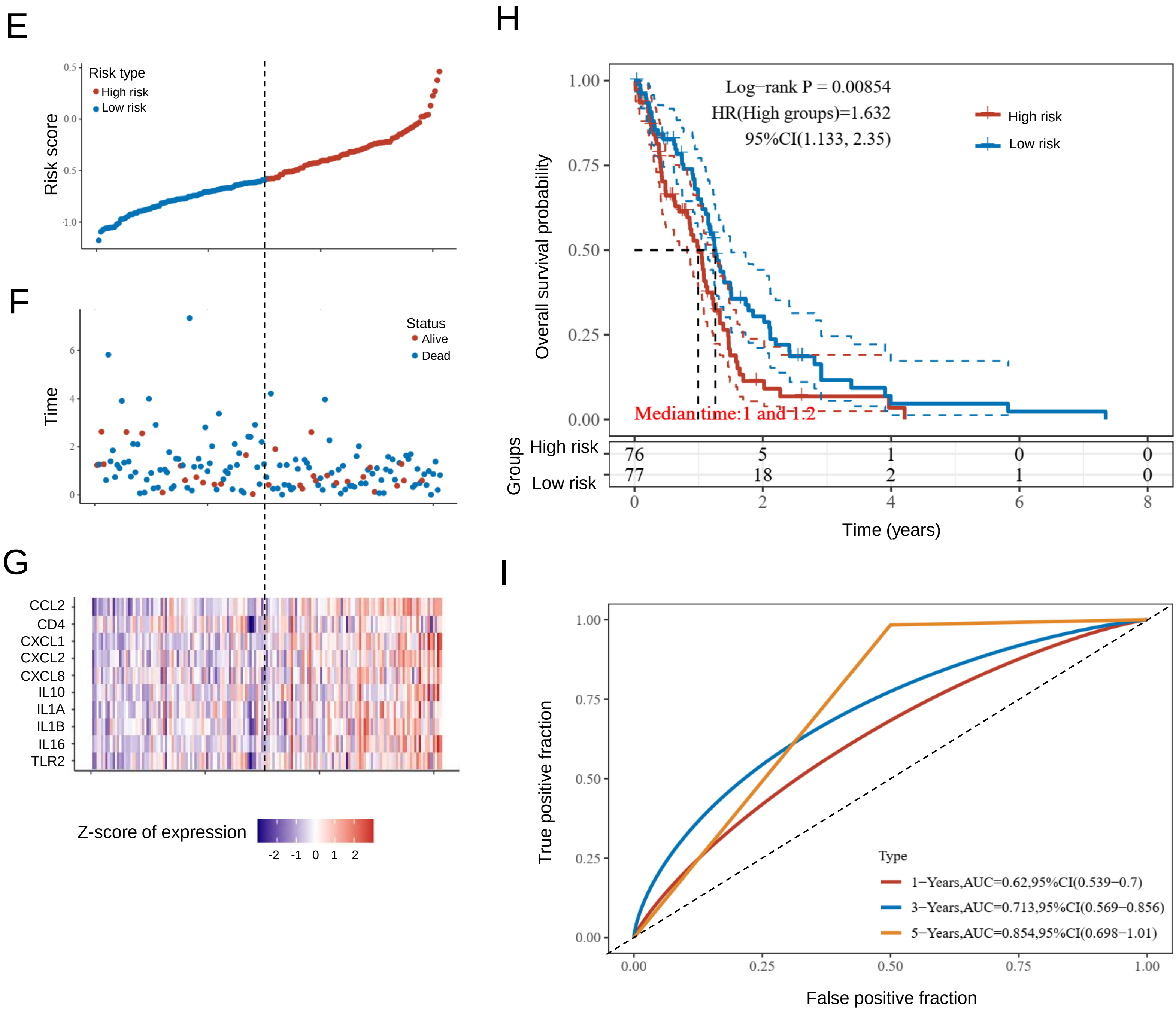

Fig. S5 Functional analysis of CCL2 and associated genes. **A-C** Three genes representative of 370 genes which are most positively associated with CCL2 in GBM in the TCGA dataset. r, Spearman's correlation coefficient. **D** The expression of 10 hub genes in GBM and normal counterparts was compared using the TCGA database. TPM, transcripts per million. Student's T-test followed by the Benjamini-Hochberg false discovery rate correction was used to define the threshold of significance. Distribution of the risk score in the TCGA training set. **F** The survival time and survival status between high- and low-risk groups. **G** Heatmap of the expression profiles of 10 hub genes in low- and high-risk groups. The dotted line indicated the median risk score and divided the cohort into low- and high-risk group. **H** Kaplan-Meier survival analysis of the overall survival (OS) of GBM patients in the high- and low-risk groups. **I** Time-dependent ROC analysis of the predictive efficiency of this 10-gene signature on the 1-, 3-, and 5-years OS rate. *** $p < 0.001$

Fig S6

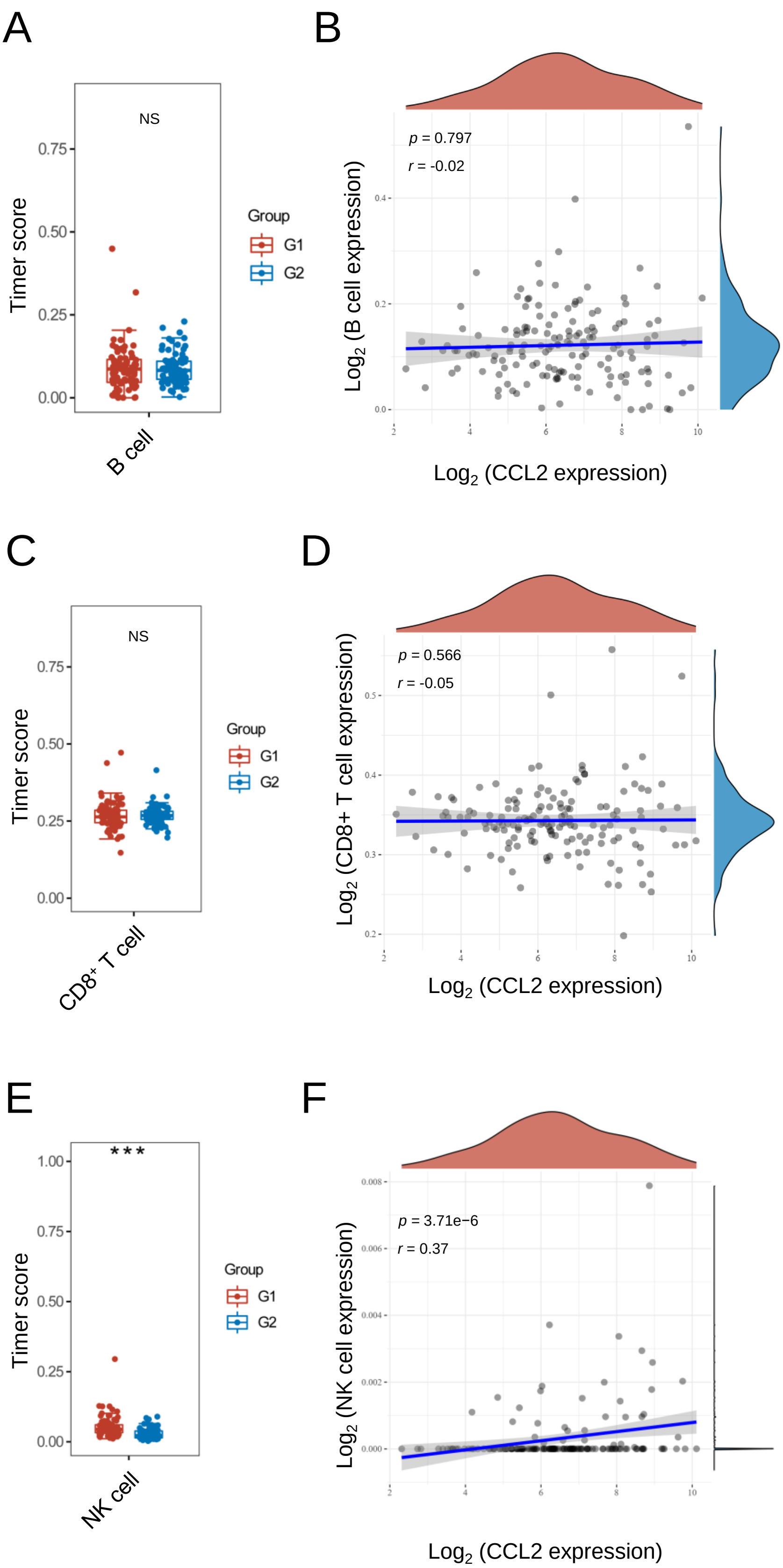

Fig. S6 Correlation between immune cell infiltration and CCL2 in GBM. **A** Immune cell score of B cells in CCL2-high (G1) and CCL2-low (G2) GBM tissue was calculated with the TIMER2.0 algorithm. **B** The correlation between CCL2 expression and immune score of B cells was analyzed with Spearman's correlation analysis. **C-F** Similar analyses for $CD8^{+}$ T (**C**,**D**) and NK (**E**, **F**) cells were performed and results were displayed as in A and B. The statistical difference was calculated through the Wilcox test. *** $p < 0.001$;
NS, not significant

Fig S7

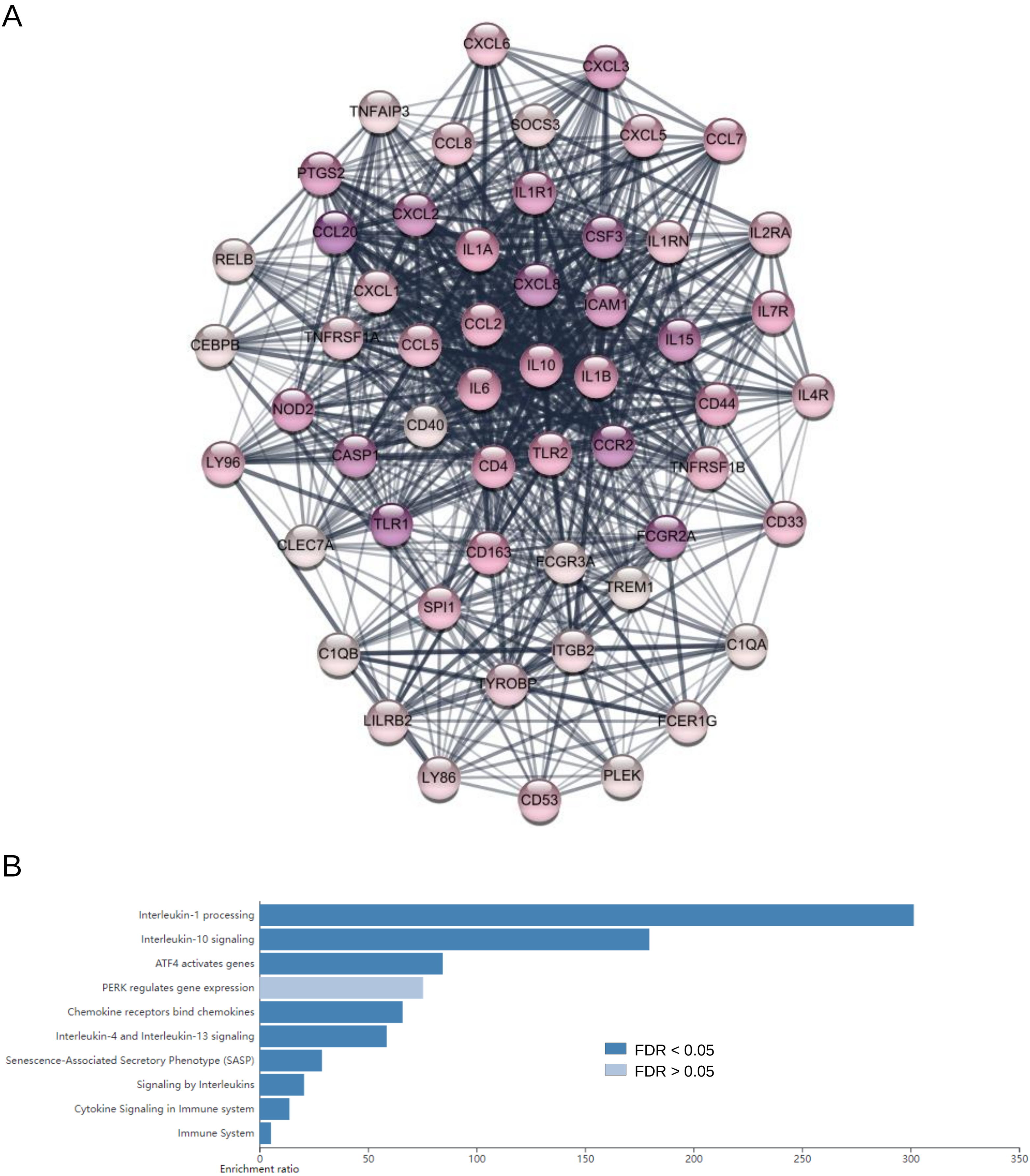

Fig. S7 Functional analysis of CCL2 in GBM. **A** The PPI network associated with CCL2 was constructed by Cytoscape software. **B** Functional analysis of reactome of 10 hub genes was performed using the online toolkit WebGestalt.
